# Schlemm's canal: the outflow ‘vessel’

**DOI:** 10.1111/aos.15027

**Published:** 2021-09-13

**Authors:** Katarzyna Lewczuk, Joanna Jabłońska, Joanna Konopińska, Zofia Mariak, Marek Rękas

**Affiliations:** ^1^ Department of Ophthalmology Military Institute of Medicine Warsaw Poland; ^2^ Department of Ophthalmology Medical University in Bialystok Białystok Poland

**Keywords:** aqueous humour, channelography, conventional drainage pathway, glaucoma, outflow resistance, Schlemm's canal

## Abstract

In a healthy eye, the aqueous humour (AH) flows via the ciliary body and trabecular meshwork into the collector channels, which carry it to the episcleral veins. In glaucoma, a heterogeneous group of eye disorders affecting approximately 60 million individuals worldwide, the juxtacanalicular meshwork offers greater resistance to the outflow of the AH, leading to an increase in outflow resistance that gradually results in elevated intraocular pressure (IOP). The present review comprehensively covers the morphology of Schlemm’s canal (SC) and AH pathways. The path of the AH from the anterior chamber through the trabeculum into suprascleral and conjunctival veins via collector channels is described, and the role of SC in the development of glaucoma and outflow resistance is discussed. Finally, channelography is presented as a precise method of assessing the conventional drainage pathway and facilitating localization of an uncollapsed collector and aqueous veins. Attention is also given to the relationship between aqueous and episcleral veins and heartbeat. Possible directions of future research are proposed.

## Introduction

Glaucoma is a leading cause of irreversible blindness worldwide and results from damage to the optic nerve (Quigley & Broman 2006; Wang et al. 2017). Although the pathology is located on the fundus of the eye, namely, loss of retinal nerve fibres and ganglion cells, this process originates in the anterior chamber, specifically in Schlemm’s canal (SC). A few available antiglaucoma therapies target the site of the pathology (SC), whereas the great majority aim to decrease intraocular pressure (IOP) by other mechanisms, either reducing aqueous production or diverting aqueous flow through the unconventional outflow system. Understanding these mechanisms and the morphology of SC, which is a unique, complex vascular structure responsible for maintaining fluid homeostasis in the eye, is crucial for therapeutic decisions.

Aqueous humour (AH) drains from the eye via two physiological pathways. The conventional path begins at the level of the irido‐corneal trabecular meshwork (TM) and is responsible for approximately 83–96% of drainage (Dautriche et al. 2015). Aqueous humour (AH) flows from the anterior chamber through the TM into SC, followed by passage into the collector channel (CC) along the SC external wall. From the CC, aqueous‐containing vessels extend outward to discharge into visible episcleral and conjunctival veins on the scleral surface (Xin et al. 2017). The remaining 4–17% of flow may leave the eye through the uveoscleral, or unconventional, outflow pathway, which involves passive fluid movement down a pressure gradient (Fautsch & Johnson 2006). It is unclear whether this percentage changes with age or stress (Fautsch & Johnson 2006). In uveoscleral outflow, AH enters the ciliary muscle and exits through the supraciliary space, moving across the anterior or posterior sclera through the emissarial canals around the vortex veins, or into the choroidal vessels (Johnson & Erickson 2000; Bhartiya et al. 2015). Uveoscleral outflow may be considered analogous to lymphatic drainage of tissue fluid, as the fluid may mix with tissue fluid from the ciliary muscle, ciliary processes and choroid and is drawn osmotically into veins (Bhartiya et al. 2015; Johnson et al. 2017).

Aqueous humour (AH) flows out of the anterior chamber as a mass stream regulated by a basal‐to‐apical pressure gradient (Parc & Johnson 2003). As AH from the anterior chamber fills SC, pressure must be lower than that in the anterior chamber to permit flow. The reduction in SC pressure that allows entry into the SC lumen simultaneously requires a one‐way mechanism to prevent AH backflow into SC from the episcleral veins. Moreover, pressure in the episcleral veins is normally lower than that in the CC, and the mean pressure in the CC must be lower than the pressure in SC to permit AH flow. In healthy human eyes, outflow facility has a value of 0.40 µl at 10 mmHg and decreases with age (Parc & Johnson 2003). The average rate of production of AH is 2.0–2.5 µl/min, and the turnover rate for aqueous volume is approximately 1% per minute (Andrés‐Guerrero et al. 2017). From a physiological perspective, the trabeculum, particularly the interior wall of SC, and the TM near the CC are the main sources of resistance to aqueous outflow, and the remaining part of resistance is located in the exterior wall and surrounding tissues (Gabelt et al. 2011; Kiland et al. 2011). This area is called the juxtacanalicular space and is assumed to be the primary site of IOP regulation (Goel et al. 2010). Elevated IOP in glaucoma is caused by an increase in AH outflow resistance on its drainage pathways and not by an increase in AH production (Kagemann et al. 2014b). Overall, outflow resistance is not constant but rather a function of IOP and increases as IOP rises.

## Scientific Background

Aqueous humour (AH) flows out of SC through one of around 30 CCs and aqueous veins and then to the system of suprascleral veins, ophthalmic veins and general circulation (Alvarado et al. 2005a). According to Poiseuille's law, the resistance of aqueous veins should be insignificant if they are not collapsed or compressed. Provocative gonioscopy, during which blood reflux into SC is observed, is the simplest method of assessing the conventional drainage pathway and facilitating localization of an uncollapsed collector and aqueous vein. This technique permits direct observation of the TM; indeed, the transparency of the TM enables easy observation of blood entry into SC. Assessment of the distribution of aqueous veins in channelography is a more precise method (Movie S1). A study by Grieshaber (2015) showed a relationship between postoperative IOP and the presence of reflux in SC before surgery and between the degrees to which water veins filled. These authors assessed transtrabecular diffusion by channelography and the filling properties of the episcleral venous system by a microcatheter and a fluorescein tracer placed in SC during canaloplasty. The analysis revealed that blood reflux varies greatly in glaucomatous eyes, with an inverse correlation with preoperative IOP: The higher is the IOP, the poorer is the blood reflux. The filling qualities of the episcleral venous system and diffusion through the TM also differed in different glaucoma stages. The researchers concluded that poor trabecular passage and good episcleral fluorescein outflow indicate patent distal outflow pathways, that poor trabecular passage and poor episcleral fluorescein outflow indicate an obstructed TM and closed CCs, and that good trabecular passage together with poor episcleral fluorescein outflow suggest that the site of impairment is mainly in the distal outflow system. In normal subjects, the pressure gradient reversal causes SC filling to begin in 5–10 seconds and to finish in 15–30 seconds (Carreon et al. 2017). A similar rapid elimination of SC blood restores normal pressure gradients (Schirmer 1971). Initially, SC fills rapidly and completely (Schirmer 1969).

In ocular hypertension, rapid SC filling slows, even though the canal eventually fills with minimal impairment to the outflow facility (Suson & Schultz 1969). As glaucoma progresses with deteriorating outflow facility, filling defects appear, and the SC no longer fills completely with blood (Suson & Schultz 1969). In more advanced glaucoma, SC blood reflux fails to occur, even when aggressive measures are implemented to reverse pressure gradients (Kronfeld & Haas 1944).

Zhou et al. (2012) conducted a study in which SC cell mechanical properties that may modulate AH outflow resistance were examined. The authors supported the hypothesis that mechanical properties of the SC endothelium may contribute to AH outflow resistance, presumably through effects upon modulation of pore formation. Schlemm’s canal (SC) cells are highly contractile and responsive to a range of pharmacological interventions, and it was found that drugs known to increase outflow resistance cause SC cells to stiffen and, conversely, that drugs known to decrease outflow resistance cause SC cells to soften. These responses varied among patients (Zhou et al. 2012).

In addition, Battista et al. (2008) used bovine eyes to study the effects of increased IOP in the range of 7–45 mmHg on SC, showing that increasing IOP coincides with a twofold reduction in effective AH outflow. Additionally, increasing IOP to 45 mmHg caused a progressive collapse of SC, and its walls herniated into the outlet of the aqueous veins, leading to further outflow obstruction; 95% of collectors were also blocked when IOP >30 mmHg. These observations were confirmed in another study by Grieshaber, which showed that the number of closed collectors increased at IOP higher than 20 mmHg; at IOP exceeding 25–30 mmHg, most of the CCs were closed, and SC collapsed (Grieshaber et al. 2010).

## Anatomy of Schlemm's Canal

**Fig. 1:**
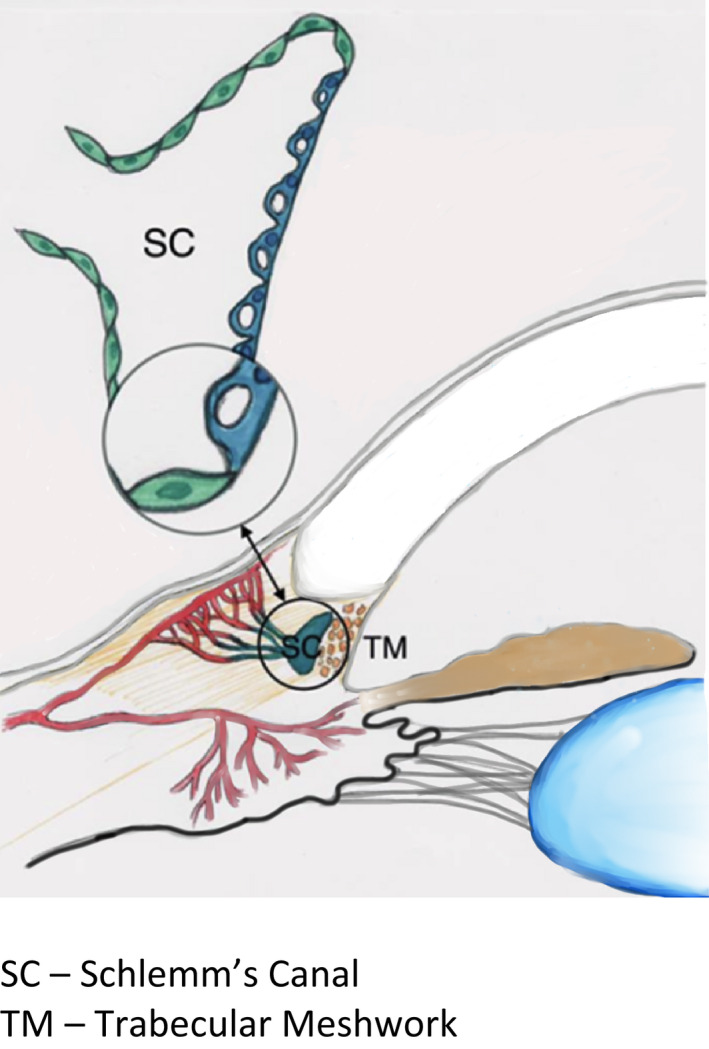
The anatomy of Schlemm’s canal.

**Fig. 2:**
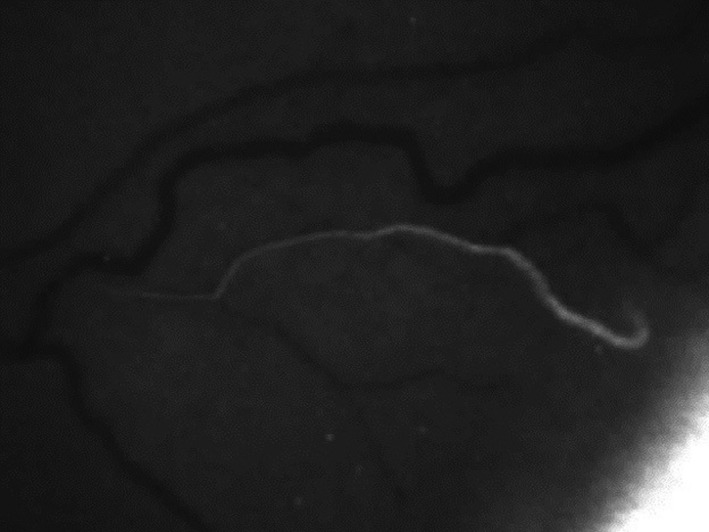
The aqueous vein visible in channelography.

**Fig. 3:**
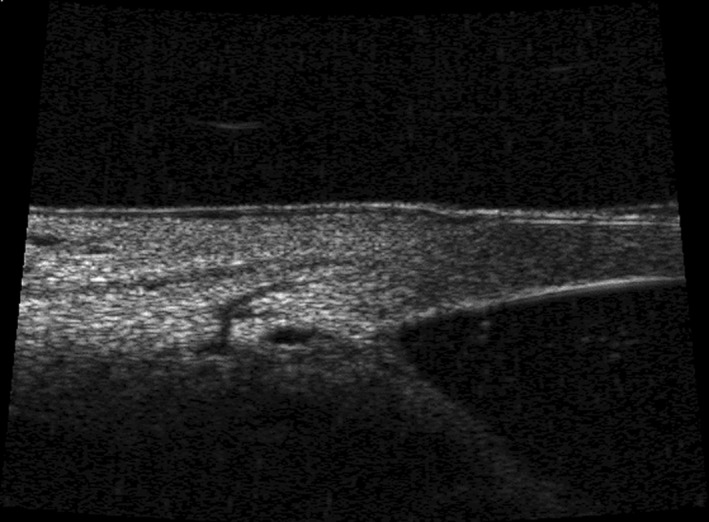
SC – (Schlemm’s canal) and CC (collector channel) visible on ultrasound biomicroscopy images.

Schlemm’s canal (SC) was named in honour of the German anatomist Friedrich Schlemm, who in 1830, discovered the canal in the anterior chamber angle, whereby AH entered into the bloodstream (Dvorak‐Theobald 1955; Mansouri & Shaarawy 2015). It is a ring‐like canal with a length of 36–40 mm encircling the cornea (Parc & Johnson 2003; Byszewska et al. 2019) and lies directly adjacent to the juxtacanalicular trabecular meshwork (JCT) (Dautriche et al. 2015); it forms the conventional outflow pathway together with the TM (Goel et al. 2010; Truong et al. 2014) (Fig. 1). In cross‐section, SC has the shape of an elongated ellipse, with its longer axis measuring 150–350 µm. 3D visualizations have enabled precise measurements of the canal, and the cross‐sectional area of which ranges from 4064 to 7164 μm^2^ (Kagemann et al. 2012, 2014a, 2014b) (Figs 2 and 3). Rarely, the canal may be bi‐ or tripartite (Ten Hulzen & Johnson 1996), and it may sometimes contain septa (Dietlein et al. 2000). One of the primary functions of SC is to drain AH from the trabeculum to the CC.

Because of its location directly adjacent to the trabeculum, not all SC cells are identical (Lai et al. 2019; Vahabikashi et al. 2019). Owing to the canal's microanatomy, one can distinguish between the inner and outer walls, each built of a continuous and single‐cell layer of endothelium. The cells of both walls differ in terms of morphology (Hamanaka et al. 2016), expression of different marker, organelles and function (Karl et al. 2005) (Table 1). The inner wall is more frequently analysed because it presents the greatest resistance to drainage of AH (Vranka et al. 2015; Fan et al. 2020; Osmond et al. 2020). Endothelial cells of the inner wall are shaped like paver stones, and cells of the outer wall are smooth and flat (Ethier 2002). Tight junctions of VE‐cadherin as well as characteristic giant vacuoles and pores are markers of cells of the inner wall. Desmin, which is reactive to factor VIII‐related antigen, and the presence of Weibel‐Palade bodies are markers for cells of the outer wall (Tamm 2009; Vahabikashi et al. 2019).

**Table 1:** Comparison of external and internal wall of Schlemm’s canal

External wall	Inner wall
Slender and flat cells	Cobble‐shaped cells
Basement membrane continuity	Porous basement membrane. Presence of tight junctions
Weibel‐Palade cells	Giant vacuoles and pores
Presence of Desmin	Presence of VE‐cadherin
Reactivity with the VIII factor antigen	
Star‐shaped F‐actin systems	Circumferential F‐actin bands

## Embryogenesis

Schlemm’s canal (SC) is a highly specialized vessel. Despite many similarities to the vascular endothelium, the canal's embryonic origin and progression of its development have still not been precisely determined (Dautriche et al. 2015). Early research suggested a vascular origin (Wulle 1968; Smelser & Ozanics 1971), but recent publications have classified these cells as unique endothelial cells with phenotypical traits of both blood and lymphatic vessel endothelial cells (Aspelund et al. 2014; Karpinich & Caron 2014; Kizhatil et al. 2014). In humans, SC prenatal development begins with the development of the trabeculum in the 17th week (Ethier 2002); in the 24th week, the canal is already defined and encircles the limbus over 360°; and the canal and CCs are fully developed in the 36th week (Ramírez et al. 2004). The organogenesis of SC was described by Kizhatil as a combination of the vascular developmental factors of angiogenesis and lymphangiogenesis (Kizhatil et al. 2014). He termed this process ‘canalogenesis’, which begins from the limbal vascular plexus. The development of SC can be divided into four stages, starting from differentiation of the canal's precursor cells, proliferation and migration of frontal cells, formation of the canal's lumen, and separation from the venous vascular system (Dautriche et al. 2015; Xin et al. 2017). Prospero homeobox protein 1 (PROX1) and vascular endothelial growth factor receptor 3 (VEGFR‐3) expression are required for division of frontal cells and for shaping them into the canal.

## Genetics

Prospero homeobox protein 1 (PROX1) is the main regulator of lymphangiogenesis, and its expression is critical in transforming cells of the vascular endothelium into cells of the lymphatic endothelium (Aspelund et al. 2014; Park et al. 2014; Dautriche et al. 2015). Truong et al. (2014) were the first to demonstrate a high level of expression of the lymphatic transcription factor PROX1 in canal endothelial cells, thus showing similarity to lymphatic endothelial cells. VEGFR‐3 is a receptor belonging to the *kinase insert domen‐containing receptor* family (RTKs‐KDR); it binds the vascular endothelial growth factors VEGF‐C and VEGF‐D, and its expression is typical of the lymphatic vessel endothelium (Neufeld et al. 1999). Park and Aspelund presented the properties of the precursor cells of SC, and key molecular mechanisms required for differentiation of these cells into the mature cells of the canal (Aspelund et al. 2014; Park et al. 2014). Moreover, Aspelund demonstrated that VEGF‐C is necessary for activating the migration of vascular endothelial cells and their further formation from transscleral venous vessels. He also demonstrated that precursor cells are, in essence, vascular endothelial cells expressing VEGFR‐2 and tunica interna endothelial cell kinase (TIE2). TIE2, which is expressed almost exclusively in endothelial cells in mice, rats and humans, possesses a unique extracellular domain containing two immunoglobulin‐like loops separated by three epidermal growth factor‐like repeats that are connected to three fibronectin type III‐like repeats. The ligand for the receptor is angiopoietin 1. Defects in TIE2 are associated with inherited venous malformations; in fact, the TIE2 signalling pathway appears to be critical for endothelial cell‐smooth muscle cell communication in venous morphogenesis. Precursor cells acquire PROX1 expression to create and form the canal's lumen and VEGFR‐3 for later maturation of the canal’s cells (Aspelund et al. 2014). Both AH and VEGF‐C are required for proper SC development. A reduction in AH in mice results in the loss of elements of canal cell lymphatic identity (Park et al. 2014). The direct relationship of the SC endothelium with the JCT and the fact that the development of the TM precedes the development of SC allow for the hypothesis that soluble factors from JCT cells may be of critical significance for obtaining phenotypical traits of SC cells. Because the inner wall of SC is in direct contact with the TM over a 360° circumference, modern canal surgery provides access to the entire inner wall of the SC and the juxtacanalicular region without affecting the cornea, iris or ciliary body. Furthermore, canaloplasty may be used to deliver transgenic SC/TM vectors in glaucoma gene therapy (Tian & Kaufman 2013).

## Role of nitrogen oxide

Several studies have documented the influence of cytokines (TNF‐α, IL1‐α, IL‐β and IL‐8) released by TM cells on SC cells, as well as their influence on the regulation of AH drainage (Alvarado et al. 2004, 2005a). Nitrogen oxide (NO) has been widely studied from the perspective of its role in modulating the behaviour of SC cells and regulating AH flow (Ellis et al. 2010; Ashpole et al. 2014). Stresses in the SC endothelium trigger NO production in SC cells, similar to other vascular endothelial cells (Ashpole et al. 2014). Increased shear stress and NO production during SC collapse at elevated IOPs may in part mediate IOP homeostasis. Additionally, NO reduces SC cell volume, suggesting that the NO‐induced reduction in SC cell volume may influence outflow facility. Changes in SC cell volume in response to changes in osmolarity have also been demonstrated (Ellis et al. 2010). In general, up‐regulation of NO production may prevent proper normalization of IOP and play a role in ocular hypertension in glaucoma.

## Biomechanics

The hydraulic conductivity of the conventional aqueous drainage pathway amounts to approximately 10^−7^ cm^2^ s^−1^ g^−1^, and this value also sets the lower limit for hydraulic conductivity of the SC endothelium, which is 2–5 times greater than that of the brain endothelium and the greatest in the human body (Zhou et al. 2012). In SC, the biomechanical conditions acting on endothelial cells resemble the microenvironment of a lymphatic vessel (Kizhatil et al. 2014). In SC endothelial cells, the pressure gradient is distributed from the base to the apex of a cell, similar to lymphatic vessels but the opposite of the distribution of the vascular endothelium (Stamer et al. 2015). In a typical blood vessel, the basement membrane and surrounding tissue provide additional support for endothelial cells, reducing circumferential and radial stresses on cells. In the case of SC cells, the inverted pressure gradient caused by AH flowing into the canal’s lumen generates a force that pushes cells away from the basement membrane (Braakman et al. 2014). However, in contrast to a lymphatic vessel, SC cells are bound by tight junctions; hence, they maintain the pressure difference between the eyeball and episcleral veins. Forces related to the pressure drop from the base to the apex of a cell cause cell deformation and the formation of large, dome‐shaped diverticulae in the canal’s lumen, called giant vacuoles (Zhou et al. 2012; Chen et al. 2014; Stamer et al. 2015). In addition to tight junctions between endothelial cells, there are extensive links between endothelial cells and cells in the JCM area. These junctions are present when SC cells form protrusions to join with the JCM cells, generating parachute‐like structures. These junctions, as described by Johnstone, play an important role in anchoring the canal’s endothelial cells in response to increases in pressure (Ethier 2002; Johnstone et al. 2011). The size of the SC lumen changes in response to IOP fluctuations (Johnstone 1979). When IOP increases, the TM widens while the canal narrows; this is caused by an increase in the number of vacuoles and of the area of the extracellular matrix (ECM) as well as by the fact that both walls of the canal are close to one another. At high IOP, the probability that the canal's walls will collapse and resistance on drainage outflow pathways will grow increases significantly (Johnstone 1979). When IOP increases to approximately 40 mmHg, the canal collapses, with the exception of segments containing septa (Van Buskirk 1982; Battista et al. 2008), which support the walls of SC and prevent CC occlusion (Johnstone 1979; Van Buskirk 1982). In eyes with glaucoma, the lumen of the SC is smaller than that in healthy eyes (Yuan et al. 2016).

## Microanatomy – Giant Vacuoles and Pores

Giant vacuoles are potential spaces between the ECM and the inner wall cells of the SC (Ethier 2002). Giant vacuoles form dynamically and respond to changes in IOP instantaneously (Epstein & Rohen 1991; Dautriche et al. 2015), and their quantity and size increase as IOP increases. After enucleation, the IOP decreases to zero, and vacuoles disappear within <3 min (Parc et al. 2000). The majority of giant vacuoles are found near CC outlets (Parc et al. 2000), which suggests that a greater pressure gradient is present at these sites due to greater aqueous flow (Ethier 2002). Most likely due to the specific biomechanical microenvironment, endothelial cells are characterized by contractile properties and by an elastic modulus of 1–3 kPa (Zhou et al. 2012), which is slightly greater than that of other endothelial cells (Zeng et al. 2010; Stamer et al. 2015). In addition, SC cells have the capability to adapt to deformations to the cytoskeleton system fortified with actin microfilaments. Cells of the outer wall have star‐shaped F‐actin systems that pass through most cells, in contrast to the circumferential F‐actin bands observed in endothelial cells of the inner wall (Ethier et al. 2004). The position of SC cells relative to the ECM allows for the reception of biomechanical signals from the matrix (Alvarado et al. 2005b), which models the expression of genes and adapts them to changes in substrate rigidity. Overall, the rigidity and contractility of SC cells exhibit a strong response to pharmacological stimulation. Medications that increase resistance to drainage enhance the rigidity of SC cells; conversely, medications that reduce resistance to drainage decrease the rigidity of these cells (Zhou et al. 2012). In eyes with glaucoma, endothelial cells are more sensitive and exhibit an amplified response to the increase in the substrate's rigidity that occurs with the disease (Stamer et al. 2015). Furthermore, stress caused by a rise in IOP can increase cell surfaces by up to 50% and even cause them to thin (Hamanaka et al. 1992). Tight junctions between endothelial cells of the inner wall are very sensitive to increases in IOP and become less complex when IOP is elevated (Alvarado et al. 2005a). Endothelial deformation may initiate the formation of pores mediating aqueous transport by loosening intercellular junctions (Johnson et al. 2002; Tamm 2009).

Pores, structures in the inner wall with sizes ranging from 0.6 to 3 µm (Ethier 2002; Braakman et al. 2014), are responsible for 10% of the resistance to AH drainage (Alvarado et al. 2004). Such pores may be found in the walls of giant vacuoles, but they may also be unrelated to them (Tamm 2009); they also form the main pathway of aqueous flow through the inner wall of SC. Two pore types have been identified and characterized, type I pores (transcellular) and type B pores (paracellular) (Ethier et al. 1998), which differ in their locations, filtration ability and formation mechanisms (Braakman et al. 2014). Type B pores are larger, but they are outnumbered 3‐4 to 1 by type I pores. Type B pores form as a result of local loosening and widening of intercellular junctions (Braakman et al. 2014). Braakman presented a segmentation of the aqueous drainage stream, with type B pores accounting for the majority of aqueous flow (Braakman et al. 2014). Type I pores may form as a result of a combination of deformations of the cellular membrane at the base and apex of an endothelial cell, which may occur under the influence of the aqueous filtration stream and caveolae, vesicles and minipores (Herrnberger et al. 2012; Braakman et al. 2014). Pores in SC are most frequently formed from minipores 60 nm in size and are covered by a diaphragm containing plasmalemma vesicle‐associated protein (PLVAP) (Herrnberger et al. 2012; Braakman et al. 2014). Although molecular pore formation processes are not well known, PLVAP is most likely involved, considering that pore formation is significantly impaired in mice with PLVAP deficiency (Herrnberger et al. 2012). The pore density in the interior wall fluctuates between 1000 and 2000/mm² (Johnson et al. 2002), and the number of pores in the inner wall increases when IOP is elevated (Johnson et al. 2002; Ethier et al. 2004). Giant vacuoles and pores are unique features of the endothelium of SC’s inner wall and of the endothelium of the arachnoid villi in the central nervous system (Tripathi & Tripathi 1974; Sit et al. 1997).

The formation of giant vacuoles occurs in one direction, providing a preferential aqueous drainage pathway through the endothelium by means of a one‐way valve mechanism. In the case of a pressure increase in episcleral veins and in SC that exceeds IOP, the number of vacuoles and pores decreases, preventing blood reflux from SC into the anterior chamber (Alvarado et al. 2004; Filla et al. 2011) Certain medications, such as glucocorticosteroids or sphingosine‐1‐phosphate, which induce polymerization of cytoskeleton proteins (Clark et al. 1994; Filla et al. 2011), may inhibit the formation and reduce the density of vacuoles, thereby increasing resistance to drainage (Underwood et al. 1999; Sumida & Stamer 2010). Eyes with glaucoma exhibit reduced pore density, which emphasizes the critical role of the inner wall in maintaining AH homeostasis. Aqueous flow resistance is considerably increased by the hydrodynamic interaction between pores and their basal substrate, the subendothelium (basement membrane of SC cells and JCT ECM) (Johnson 2006). In particular, flow is concentrated near every pore, forming funnels that pass through the region of the ECM closest to a given pore, which significantly reduces the effective area available for flow through these regions (Stamer et al. 2015). It is still unknown why SC cells are morphologically heterogeneous: some cells form giant vacuoles, whereas others are flat. It might constitute a cellular phenomenon or relate to aqueous flow pathways upstream (Fautsch & Johnson 2006). The goal of glaucoma therapy oriented towards SC may be to increase pore density and thus drainage, leading to a reduction in IOP (Johnson et al. 2002).

## Distribution of Aqueous Humour

Aqueous humour (AH) in SC is not distributed uniformly through the canal's inner wall but rather appears preferentially at certain locations. Drainage of AH most frequently occurs near the CC (Hann & Fautsch 2009). In fact, twice as many giant vacuoles are present near collectors, which suggests that aqueous flow through the inner wall is dependent on the pressure value (Parc et al. 2000). Studies involving the application of fluorescent markers have also demonstrated an elevated level of markers in the pigmented part of TM adhering to the CC, suggesting that the preferred drainage outflow pathways are present near collectors (Hann & Fautsch 2009). According to histological research on human eyes, CCs are randomly distributed around the eye between the 25th and 30th year of life, with preferential dislocation in the inferior nasal quadrant (Rohen & Rentsch 1968). This has been confirmed by 3D micro‐CT tests (Hann et al. 2011). Regardless, there is high diversity in the size of CC outlets, with values ranging between 550 µm and up to 70 µm depending on the type of test (Rohen & Rentsch 1968; Hann et al. 2011). From the CC, AH flows through a winding system of venous plexuses, from the deep scleral plexus, through the limbal plexus, to the intrascleral plexus, which ultimately leads to the episcleral veins (Goel et al. 2010).

### Aqueous veins

**Fig. 4:**
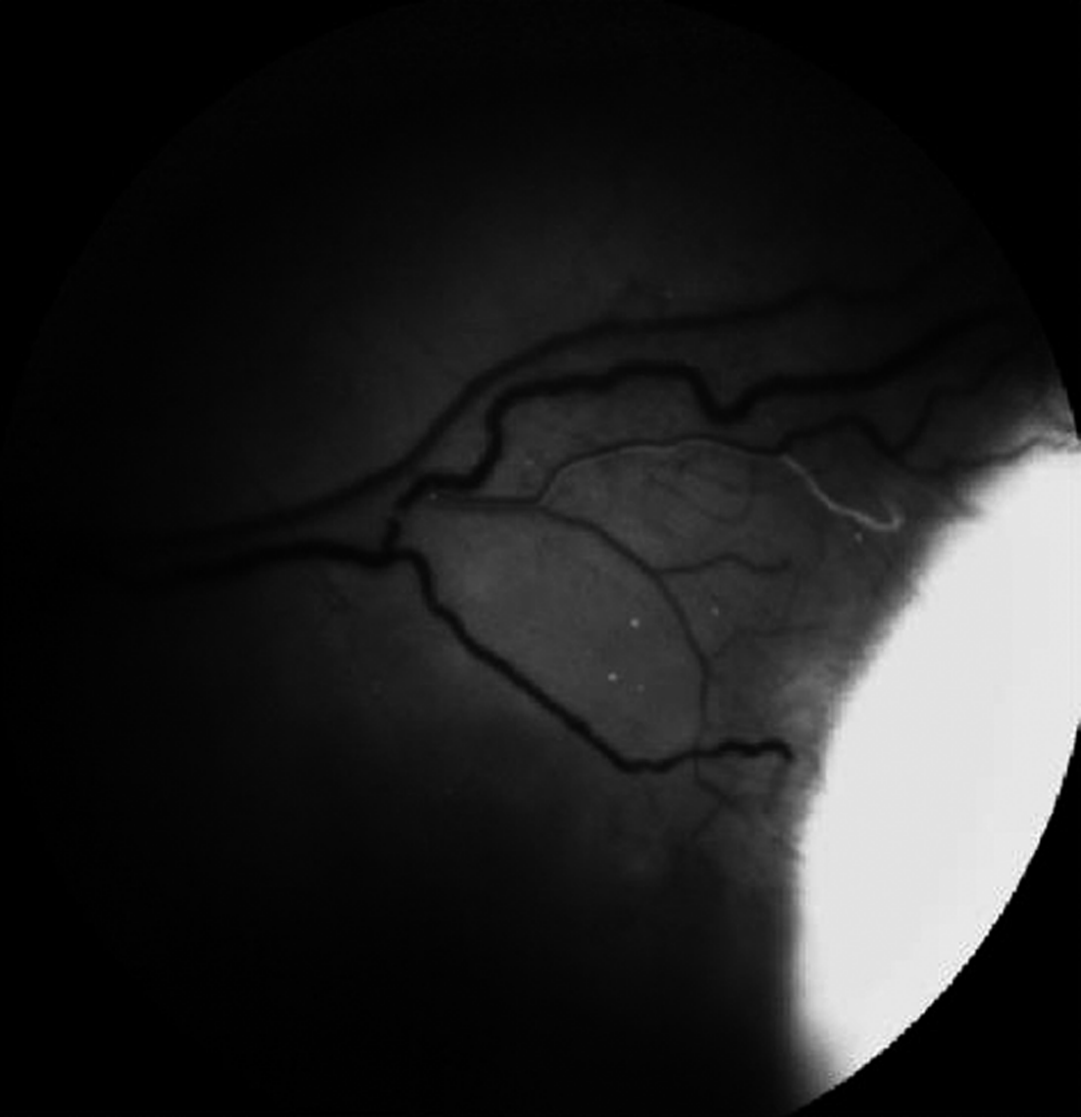
Aqueous veins.

**Fig. 5:**
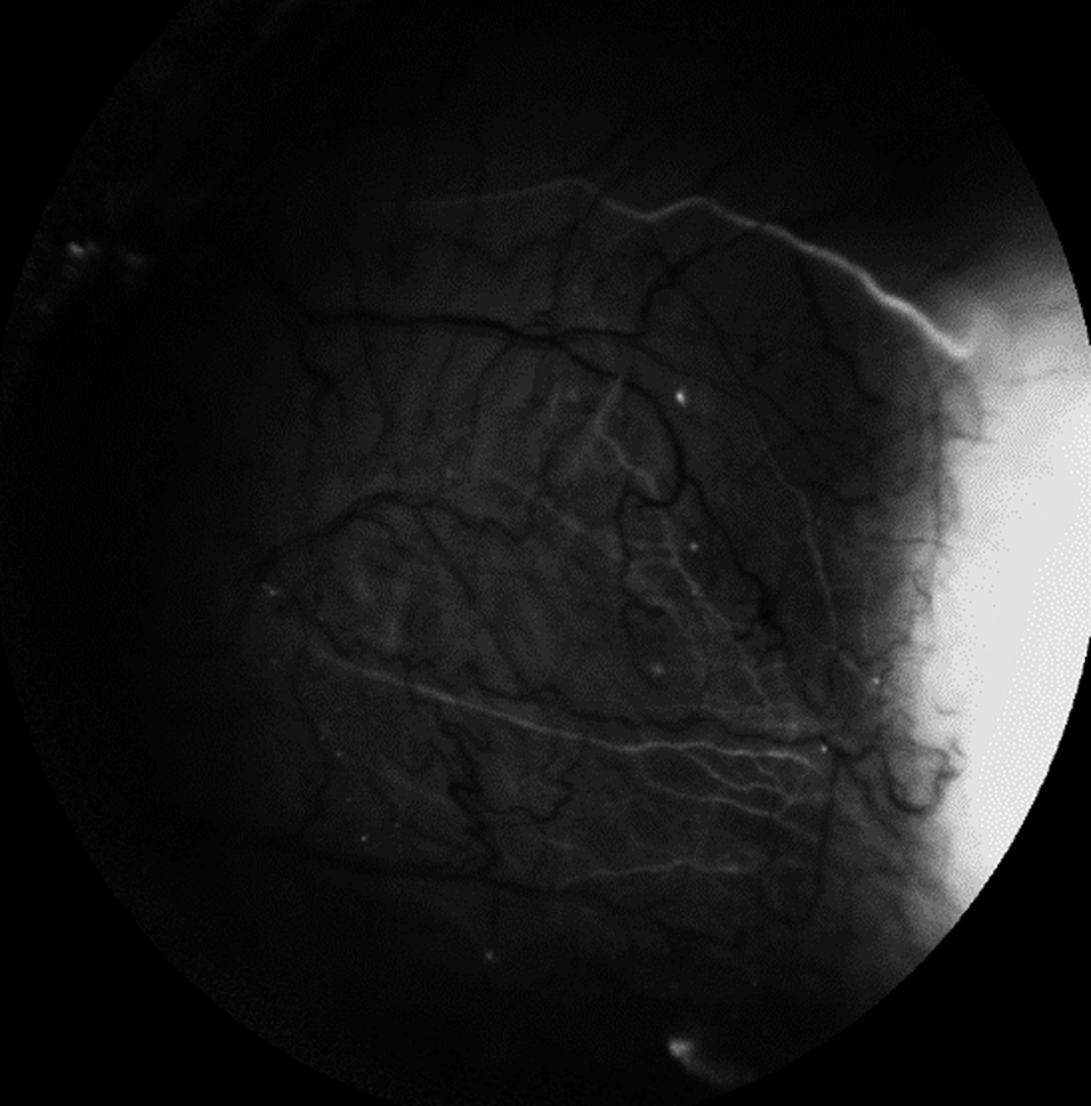
Episcleral veins.

Aqueous humour (AH) moves through the TM into SC and flowing from its lumen into the CC, aqueous veins (AVs) (Fig. 4) and the system of episcleral veins (EPVs) (Fig. 5), ocular veins and into the general circulation (Tamm 2009). AVs have lumens that are directly connected to the CC, and thus, they are directly connected to the episcleral veins draining blood into the general circulation, bypassing the deep scleral and intrascleral venous plexuses (Ascher 2018). AVs containing initially clean AH are joined to episcleral veins filled with blood, which is why transition zones can be observed on the surface of the conjunctiva as large vessels with a transparent, central lumen bounded by dark blood from all sides. Linear stratification into AH and blood occurs due to differences in the viscosity and density of these fluids (Meighan 1956); the composition of blood and AH in transition zones also changes with IOP. Indeed, direct observation of these changes is a reliable indicator for assessing the effectiveness of topical and surgical therapy oriented towards IOP reduction in glaucoma (Johnstone 2004). AVs differ in their position, size and anatomical configuration. In a slit‐lamp test, 2‐3 AVs are usually visible, and up to 6 AVs may be seen (Stepanik 1954). AVs are non‐uniformly distributed and are present in the greatest number in the inferior nasal quadrants (Stepanik 1954). Their size varies from 20 to 100 µm, with an average of 50 µm. Histologically, AVs cannot be distinguished from conjunctival and EPV (Stepanik 1954).

### Aqueous humour drainage

Intraocular pressure (IOP) is the primary factor affecting AH drainage. Drainage through the conventional outflow pathway is directly proportional to IOP within the range of physiological values (Morrison & Freddo 2003). Drainage facility is the measure of how easily AH leaves the eye, and it is the inverse of resistance to drainage. In healthy human eyes, the drainage facility has a value of 0.40 µl/min/mmHg at an IOP of 10 mmHg (Brubaker 1975). The main point of resistance to AH drainage is at the JCM level in juxtacanalicular connective tissues, in the inner wall of SC and its basement membrane (Johnson 2006). Elevated IOP in glaucoma is caused by an increase in aqueous outflow resistance on its drainage outflow pathways and not by an increase in AH production (Grant 1951). Aqueous humour (AH) flow, defined as the movement of AH from the posterior chamber of the eye through the pupil into the anterior chamber, is lower than aqueous production because it does not include the AH that leaves the posterior chamber via other pathways (Grant 1951). The value of AH flow through the anterior chamber is not dependent on sex (Brubaker 1982). Aqueous humour (AH) flow is 2.4 ± 0.6 µl/min and decreases with age by 2% per decade (Alvarado et al. 2004), which may result in a reduction of up to 30% (Reiss et al. 1984). It has also been observed that flow is half at night (1.13–1.6 µl/min) compared with that during the day (3.0–3.1 µl/min) (Reiss et al. 1984; Viggiano et al. 1994). In studies using fluorescein, it was observed that the flow value is also significantly lower in eyes with pseudoexfoliation syndrome than in physiologically correct eyes (Brubaker 1982).

### Effective filtration areas

Based on observations of the distribution of pigment and perfusion markers, it was determined that circumferentially, drainage of AH in healthy eyes is non‐uniform and segmented (Battista et al. 2008; Lu et al. 2008; Cha et al. 2016): at any given time, only some AH drainage pathways are actively involved in aqueous percolation. These active areas are called effective filtration areas (EFAs) (Cha et al. 2016), and their assessment is a valuable method for measuring resistance to flow and the effects of IOP changes. Segmented drainage has been described in mice (Swaminathan et al. 2013), pigs (Keller et al. 2011), cows (Lu et al. 2008), monkeys (Lu et al. 2011) and humans (Keller et al. 2011; Yang et al. 2013). Higher marker concentration is present in the TM neighbouring the outlets of CCs, and in humans, more pigment is observed at this location, suggesting that EFA locations can be determined by using pigment distribution as a marker (Keller et al. 2011). A sudden increase in IOP in cow eyes caused a significant reduction in EFA (Battista et al. 2008). When IOP increased suddenly, the marker was present in a greater concentration near CC outlets. When IOP was correct, the drainage patterns were more uniform; when IOP was elevated, drainage became more segmented (Battista et al. 2008). Effective filtration areas (EFA) reduction is linked to a reduction in drainage facility and is reversed when pressure is reduced from high to normal levels (Zhu et al. 2013). EFA reduction has also been detected in eyes with glaucoma in an animal model and in chronic IOP elevation after laser therapy (Zhang et al. 2009). Specifically, reduction in the marker level was determined in regions of the TM that had undergone laser therapy in this study, and it was stated that active drainage shifted from areas that had undergone laser therapy to areas not affected by the therapy. In a study where a marker was applied, significant EFA reduction was observed in eyes with glaucoma in comparison with healthy eyes (Zhang et al. 2009). In addition, the inversely proportional dependence between EFA and IOP has been documented in the eyes of a mouse with ocular hypertension (Swaminathan et al. 2013).

### Pulsatile flow

In addition to the traditional approach, according to which AH moves passively in a combined stream through the TM into SC downwards along the pressure gradient determined by the heart (Bill 1975), a significant effect of the active process driven by means of a mechanical pump is also assumed (Johnstone 2004). Aqueous and suprascleral veins oscillate according to heartbeat (Johnstone et al. 2011), and these oscillations enable continuous lamellar flow (Movie S2). Pulsating flow occurs as a result of oscillating compressive force caused by transitional IOP increases occurring during the cardiac cycle as well as blinking and eye movements. These transitional IOP spikes cause microscopic deformations of the flexible structural elements of drainage outflow pathways. During contraction, the canal’s endothelial cells move to the outside, forcing AH flow towards the outlets of CCs and AVs. When the value of IOP drops, the flexible elements move back to their original configuration, which leads to a relative reduction in SC pressure, inducing AH flow into the SC's lumen (Johnstone et al. 2011). Pressure in AVs is sufficiently high and enables reverse lamellar flow from suprascleral veins at cardiac diastole; at cardiac systole, pressure in AVs increases and reverses the direction of aqueous flow with simultaneous blood reflux. The ocular pulse arises through changes in the choroidal vascular volume as the cardiac pulse oscillates between diastole and systole. These choroidal volume changes are characterized as a choroidal piston (Phillips et al. 1992). The ocular pulse can induce pulsatile TM motion outward into SC, causing a decrease in total volume in the SC lumen and a transient increase in SC pressure, allowing the increase in IOP to elicit a pulse wave of AH to leave SC (Johnstone et al. 2011).

Pulsatile flow requires a chamber, a reservoir, mobile tissue within the reservoir walls, and valve‐like inlets and outlets that utilize cyclic force to generate motion (Levick & Michel 2010).

The SC lumen functions as a chamber. The TM serves as a mobile chamber wall as it distends into SC, causing SC lumen dimension changes (Lee & Grierson 1975). Pulsatile aqueous outflow requires tissue organization at the SC inner wall endothelium to provide one‐way flow of AH into SC.

Pulsatile flow increases markedly in normal subjects, though the same pressure causes pulsatile flow to decrease or stop altogether in glaucomatous eyes. Furthermore, the pressure necessary to stop pulsatile flow correlates with glaucoma severity (Kleinert 1951). The theory that pulsating flow drives AH drainage is reflected in the dynamic equilibrium between AH and blood in AVs (Johnstone et al. 2011). During contraction, the pulse wave causes flow of AH through AVs, resulting in visible widening of the aqueous layer in their lumens (Johnstone et al. 2011). Eyes with glaucoma exhibit reduced pulsating flow in comparison with healthy eyes (Kleinert 1952). In healthy eyes, the TM is susceptible to deformation under the influence of naturally occurring, dynamic changes in pressure and volume of AH flow from the anterior chamber to SC. Overall, the reduction in pulsating flow in glaucoma may be caused by changes in TM elasticity (Wang et al. 2017).

## Conclusions

The elevated IOP in primary open‐angle glaucoma is caused by an increased resistance to the outflow of AH from the eye. However, despite over 140 years of investigation, the precise site of this generation of flow resistance remains poorly understood. Nonetheless, efforts have been made to investigate the possible causes of this pathology. The main drawback of current antiglaucoma medications is that they reduce IOP either by decreasing the synthesis of AH or by enhancing the unconventional outflow facility. Although these treatments lower IOP and slow the progression of ganglion cell damage and associated vision loss, in most cases, they do not stop it. At present, there is currently no drug treatment in clinical use that directly targets the increased flow resistance, which is a main reason for ocular hypertension in glaucoma, largely because the mechanism of increased flow resistance remains unclear. However, the new evidence shows that actin cytoskeleton‐modulating signals are involved in aqueous outflow regulation. Rho‐associated protein kinase (ROCK) is activated by some bioactive factors in the AH. Rho‐ROCK signalling regulates a cellular events, such as cell adhesion, differentiation, motility, proliferation and apoptosis. ROCK inhibitors directly affect the TM and SC, and lowers IOP by regulation of contractile properties, fibrotic activity, and permeability of the TM and SC tissues, influencing ECM production. The TM is affected earlier and more strongly than ciliary muscle cells by ROCK inhibitors. Rho‐associated protein kinase (ROCK) inhibitors also interfere with tight junctions, leading to F‐actin depolymerization, change intracellular calcium level and increasing SC‐cell monolayer permeability. Moreover, ROCK inhibitors have also shown several additional effects, including increased retinal blood flow, direct protection of neurons against various types of stress and regulation of wound healing; these benefits may potentially be useful in glaucoma treatment.

For surgical treatment, there is a trend towards a more physiological approach with regard to resistance to aqueous outflow. Exploring and targeting these biophysical changes will allow the development of new therapies for glaucoma. As a new strategy for the management of elevated IOP, therapies that act on the conventional AH outflow route should be sought.

## Supporting information

Movie S1. Assessment of the distribution of aqueous veins in channelography.

Movie S2. Oscillation of aqueous and suprascleral veins according to heartbeat.
